# 1B/(−)IRE DMT1 Expression during Brain Ischemia Contributes to Cell Death Mediated by NF-κB/RelA Acetylation at Lys310

**DOI:** 10.1371/journal.pone.0038019

**Published:** 2012-05-29

**Authors:** Rosaria Ingrassia, Annamaria Lanzillotta, Ilenia Sarnico, Marina Benarese, Francesco Blasi, Laura Borgese, Fabjola Bilo, Laura Depero, Alberto Chiarugi, Pier Franco Spano, Marina Pizzi

**Affiliations:** 1 Department of Biomedical Sciences & Biotechnologies, School of Medicine, University of Brescia, Brescia, Italy; 2 Division of Pharmacology, Department of Biomedical Sciences & Biotechnologies and National Institute of Neuroscience-Italy, School of Medicine, University of Brescia, Brescia, Italy; 3 Department of Pharmacology, University of Florence, Florence, Italy; 4 Laboratory of Chemistry for Technologies, School of Engineering, University of Brescia, Brescia, Italy; 5 IRCCS S.Camillo Hospital, Venice, Italy; Nathan Kline Institute and New York University School of Medicine, United States of America

## Abstract

The molecular mechanisms responsible for increasing iron and neurodegeneration in brain ischemia are an interesting area of research which could open new therapeutic approaches. Previous evidence has shown that activation of nuclear factor kappa B (NF-κB) through RelA acetylation on Lys310 is the prerequisite for p50/RelA-mediated apoptosis in cellular and animal models of brain ischemia. We hypothesized that the increase of iron through a NF-κB-regulated 1B isoform of the divalent metal transporter-1 (1B/DMT1) might contribute to post-ischemic neuronal damage. Both in mice subjected to transient middle cerebral artery occlusion (MCAO) and in neuronally differentiated SK-N-SH cells exposed to oxygen-glucose-deprivation (OGD), 1A/DMT1 was only barely expressed while the 1B/DMT1 without iron-response-element (−IRE) protein and mRNA were early up-regulated. Either OGD or over-expression of 1B/(−)IRE DMT1 isoform significantly increased iron uptake, as detected by total reflection X-ray fluorescence, and iron-dependent cell death. Iron chelation by deferoxamine treatment or (−)IRE DMT1 RNA silencing displayed significant neuroprotection against OGD which concomitantly decreased intracellular iron levels. We found evidence that 1B/(−)IRE DMT1 was a target gene for RelA activation and acetylation on Lys310 residue during ischemia. Chromatin immunoprecipitation analysis of the 1B/DMT1 promoter showed there was increased interaction with RelA and acetylation of H3 histone during OGD exposure of cortical neurons. Over-expression of wild-type RelA increased 1B/DMT1 promoter-luciferase activity, the (−)IRE DMT1 protein, as well as neuronal death. Expression of the acetylation-resistant RelA-K310R construct, which carried a mutation from lysine 310 to arginine, but not the acetyl-mimic mutant RelA-K310Q, down-regulated the 1B/DMT1 promoter, consequently offering neuroprotection. Our data showed that 1B/(−)IRE DMT1 expression and intracellular iron influx are early downstream responses to NF-κB/RelA activation and acetylation during brain ischemia and contribute to the pathogenesis of stroke-induced neuronal damage.

## Introduction

Cellular iron homeostasis is a finely regulated process that prevents cellular damage due to iron accumulation and the formation of free radicals through the Fenton reaction [1]. The iron concentration in the brain increases with age and is much higher in the central nervous system of subjects affected by neurodegenerative diseases [2]–[5]. An important pathogenic role of iron has been suggested in Alzheimer's, Parkinson's and Huntington's diseases, as significant iron accumulation was found in affected brain regions of patients [6]. The relevance of neuronal cellular damage by increased iron levels was further addressed by *in vitro* and *in vivo* studies of iron and 6-hydroxydopamine (6-OHDA)-dependent neurodegeneration, respectively [7]. Increased iron content, correlated with a reduced number of TH-positive neurons, was found in the substantia nigra (SN) of rats that had been overloaded with iron dextran. Significant neuroprotection was produced by deferoxamine (DFO), an iron chelator capable of permeating the blood–brain barrier, and more recent chelators in experimental models of Parkinson's and Alzheimer's diseases [8]–[12], brain ischemia-reperfusion [13], [14] and hemorrhage [15].

Iron could be transported into mammalian cells as transferrin (Tf)-bound iron (TBI) via Tf receptor (TfR) mediated endocytosis or through the non-transferrin-bound iron (NTBI) pathway via divalent metal transporter-1 (DMT1). The role of TfR-mediated iron transport in neurodegeneration and ischemia is still controversial. TBI, TBI-binding sites and TfR expression are poorly correlated with the final steady-state distribution of iron [16]. Moreover, the number of TBI-binding sites decreased in dopaminergic neurons of the SN of PD patients [17], [18], suggesting that the NTBI pathway is preferentially involved in the iron accumulation of PD brains. Conversely, both TfR and DMT1 were recently shown to increase in the ischemic cortex of rats subjected to middle cerebral artery occlusion (MCAO) [13]. A significant consensus has emerged about the involvement of the NTBI pathway in neurodegenerative diseases, with iron accumulation mediated by DMT1 in specific brain areas [19]. DMT1 is highly expressed in mammalian neuronal cells [20]–[22], [6] and is present at a relevant concentration in the basal ganglia, caudate-putamen and substantia nigra pars reticulata [23].

The mammalian DMT1 gene family (SLC11A2; Nramp2) is composed of integral membrane proteins with 10–12 putative membrane-spanning domains [24] subjected to alternative splicing. The 5′ alternative splicing of exons 1A and 1B produces the 1A and 1B DMT1 mRNA isoforms, with 1A/DMT1 predominantly expressed in kidney and duodenum and 1B/DMT1 ubiquitously expressed in the peripheral organs and brain [25]. The 3′ splicing generates two isoforms with or without the iron responsive element (IRE) motif in the 3′UTR, named (+)IRE or (−)IRE isoforms, respectively. These variants give rise to four DMT1 isoforms, all of which are active in ferrous iron transport. The two (+)IRE isoforms are post-transcriptionally regulated by the IRE/Iron Regulatory Protein (IRP) system which stabilize them in the absence of iron, while (−)IRE splice variants are not susceptible to iron regulation [25], [26], [27]. Further complexity is added by the post-translational glycosylation of DMT1, which produces two different glycosylated molecular components: the immature, partially glycosylated, endo H-sensitive form and the mature, fully glycosylated, PNGaseF-sensitive component, with molecular masses of 60 and 90 kDa, respectively [28].

A broad up-regulation of DMT1 expression was found in the substantia nigra of PD cases as well as in animal models of PD [29], [30]. However, the specific expression of (−)IRE DMT1 in neuromelanin-positive dopaminergic neurons suggested that the (−)IRE isoform is more involved in mediating abnormal increases in iron and neuronal cell loss. In line with this hypothesis, both MPP(+) treatment in dopaminergic MES23.5 cells [10] and L-DOPA treatment in primary cortical neurons produced an increase of (−)IRE DMT1 expression and increased iron content with consequent cell death. These effects were counteracted by the specific silencing of the (−) IRE isoform [31]. More recently, the role of DMT1 was highlighted as being responsible for the elevated iron content in an ischemic model of MCAO [13].

Hypoxia induces transcription factors HIF-1α and NF-κB, which, in response to various stimuli, have been found to differentially activate the 5′ regulatory regions of 1A–1B/DMT1 [32]–[36]. NF-κB was found to specifically regulate the expression of both (−)IRE and (+)IRE 1B/DMT1 isoforms in neuronal cells exposed to sodium-nitro-prusside [37], [38] and the (−)IRE DMT1 isoform in MES23.5 cells exposed to MPP+ [39]. The only indirect evidence for NF-κB-mediated DMT1 regulation in brain ischemia comes from a study showing that tanshinone IIA, a natural compound reported to inhibit NF-κB activity [40], can downregulate DMT1 expression, iron elevation and brain infarct volume in mice exposed to MCAO [13]. However, a clear-cut demonstration of DMT1 regulation by NF-κB and its role in post-ischemic brain damage is still lacking.

We recently demonstrated that post-ischemic neurodegeneration relies on the activation of the NF-κB p50/RelA dimer [41], [42] and requires specific RelA acetylation on the Lys310 residue. The acetylation status of RelA is maintained by the coordinated activity of epigenetic regulators such as the CREB binding protein (CBP), endowed with histone acetyl transferase activity, and sirtuin 1, a NAD–dependent histone deacetylase [43], [44]. The specific RelA-Lys310 acetylation discriminates neuroprotective activation of p50/RelA during brief preconditioning ischemia from p50/RelA neurotoxic activation induced by prolonged ischemia. We speculated that 1B/(−)IRE DMT1 might be an early target gene for acetylated RelA-Lys310 in the pro-apoptotic cascade activated by ischemic injury. To test this hypothesis, we investigated the expression pattern of DMT1 isoforms during the early phase of neuronal ischemia and its relationship with the acetylation status of RelA and with cell survival. Our study provides new evidence that inhibition of 1B/(−)IRE DMT1 expression, *per se* or through RelA-Lys310 hypo-acetylation, might be a potential therapeutic approach to counteract post-ischemic neurodegeneration in stroke patients.

## Materials and Methods

### Cell culture

#### SK-N-SH cell culture

The human SK-N-SH neuroblastoma cell line was purchased from American Type Culture Collection. Cells were grown as previously reported in the presence of 50 µM retinoic acid (Sigma) for 10–12 days to induce mitotic arrest and differentiation into a neuronal-like phenotype [45].

#### Primary cultures of mouse cortical neurons

Cortical neurons were prepared from 15-day embryonic mice (C57Bl/6 dams, Charles River) and cultured for 10 days as previously described [42], [46].

### Cerebral ischemia models

#### Transient middle cerebral artery occlusion (tMCAO)

All animal procedures were approved and carried out in accordance with the guidelines of Institutional Animal Care and Use Committee at University of Brescia, School of Medicine (N.07/2010), Italian Ministry of Health (n. 145/2008 – B. 30/09/2008) and the European Communities Council Directive (86/609/EEC). C57Bl/6 male mice (Harlan, Milan, Italy) were exposed to transient (20 min) MCAO as previously described [47]. Examination of infarct volume was performed in brains frozen in liquid nitrogen to avoid post-mortem changes. To prepare nuclear and cytosolic extracts, mice were killed by decapitation 4 hours after MCAO (n = 3).

Total RNA was extracted after exposure to 1 hour MCAO followed by 1 hour reoxygenation in cortices ipsylateral and contralateral to the ischemic lesion to perform expression analysis of DMT-1 mRNA isoforms by real-time polymerase chain reaction (qRT-PCR) assay (n = 3).

#### OGD

Primary cortical neurons at 11 DIV were exposed to Oxygen-Glucose-Deprivation (OGD) as previously described [42], for 3 hours. Cellular lysates were prepared at the end of OGD for the Luciferase reporter gene assay. SK-N-SH neuronal cells were exposed to 4 hours OGD for analysis of DMT1 in nuclear and cytosolic extracts. Total cellular RNA was extracted for the analysis of 5′-spliced mRNA isoforms. The cells were also grown on glass-coverslip and immunostained for (−)IRE DMT1. Total cellular lysates were analyzed for atomic iron content. SK-N-SH cells were also exposed to 8 hours-OGD and replaced in fresh DMEM without serum for 15 hours reoxygenation, with or without deferoxamine 50,100 µM. The same OGD protocol was performed at 40 hours expression of human (−)IRE DMT1 siRNA. Neuronal injury was then evaluated by measuring the amount of LDH released (Promega, Madison, WI, USA).

### Western blot analyses

Nuclear and cytosolic protein extracts were prepared for the Immunoblot analyses from differentiated SK-N-SH as previously described [42]. The following antibodies were used: anti-pan DMT1, anti-C23 nucleolin, anti-β-actin, anti-GAPDH (Santa Cruz Biotechnology, Santa Cruz, CA, USA), anti DMT1(−)IRE (Alpha Diagnostic International) and anti-human Transferrin receptor (Zymed). Normalization of protein expression was performed by densitometry analysis by Gel Pro.3 analysis software.

### Immunostaining

SK-N-SH cells were grown on poly-Lys coated cover glasses at the density of 2×10^5^ cells/2 cm^2^ in 24 wells petri dishes and differentiated as described. After 10 days, the cells were subjected to OGD for 4 hours, then fixed in paraformaldehyde in phosphate-buffered saline and incubated with Triton X-100 (0.2% in phosphate-buffered saline) containing 10% hydrogen peroxide for 15 min. Cells were blocked with Triton X-100 (0.2% in phosphate-buffered saline) containing 2%BSA, 5% goat serum, for 1 hour at room temperature and incubated for 2 hours at room temperature with polyclonal antibodies against (−)IRE DMT1 (ADI, 1∶50) or pan DMT1 (Santacruz, 1∶50). Biotinylated goat anti-rabbit immunoglobulins (1∶500, DAKO) and an ABC kit (DAKO) were used for detection following the manufacturer's instructions.

### Semi-quantitative RT-PCR and real time PCR

Total RNA was isolated with RNAeasy kit (Qiagen) from cultured cell. The first strand of cDNA was obtained by reverse transcription from 2 µg of total RNA with 0,5 µg of oligodT(23) primers (SIGMA) with the Superscript II RT (Invitrogen) according to the manufacturer's instructions, in the presence of the ribonuclease inhibitor RNasin (Promega). 10% of the reaction was used for PCR amplification by Taq DNA Polymerase (Genespin), performed at 94°C for 30 sec, 52°C for 40 sec, and 72°C for 40 sec for 20–35 cycles for β actin or DMT1 primers, respectively. The obtained cDNA was normalized against β actin by real-time PCR for every sample. The hDMT1 5′ spliced isoforms were amplified with the following primers: 1B/DMT1 For: 5′ GTTGCGGAGCTGGTAAGAATC 3′; 1B/DMT1 Rev: 5′ GGAGATCTTCTCATTAAAGTAAG 3′; 1A/DMT1For: 5′ GGAGCTGGCATTGGGAAAGTC 3′; 1A/DMT1Rev: 5′ GGAGATCTTCTCATTAAAGTAAG 3′, and human β actin For: 5′CTTCTACAATGAGCTGCGTG 3′; β actin Rev: 5′GAGGATCTTCATGAGGTAGTC 3′ in order to obtain the expected amplicon size of 222 bp. for 1B/DMT1, 290 bp. for 1A/DMT1, 320 bp. for β actin. The PCR products were resolved on ethidium bromide-stained gels and quantified by the NIH ImageJ software for densitometry. DMT1 mRNA levels were determined in triplicate in three independent experiments of 4 hours OGD versus control in SK-N-SH differentiated neuronal cells. In addition, 1A/DMT1 was amplified by RT-PCR from the immortalized human urothelial cell line URO-TSA, as a positive control, and normalized against β actin.

One microgram of total RNA extracted after exposure to 1 hour MCAO followed by 1 hour reoxygenation was retrotranscribed using iScript kit (Bio-Rad Laboratories, Hercules, CA) and amplified by real time PCR with the following mouse DMT1 isoforms specific primers: 1A/DMT1 For: 5′ AGGCTGCGCTGCTCTGAAAAGC 3′ and Rev: 5′ ATAAGAAAGCCAGGCCCCGTG 3′; 1B/DMT1 For: 5′ CAATCACGGGAGGGCAGGAG 3′ and Rev: 5′ CAATCACGGGAGGGCAGGAG 3′; (+)IRE DMT1 For: 5′ GAAAGTCCTGCTGAGCGAAG 3′, and Rev: 5′ TTGAGCACAGCCTAAGCTACAT 3′; (−)IRE DMT1 For: 5′ CGCCCAGATTTTACACAGTG 3′ and Rev: 5′ TTGGAGTGTCGGTGCTTAAA 3′, which generated amplicons of 350 bp and 385 bp for 1A- and 1B-DMT1, respectively, and 324 bp for both (−)/(+)IRE isoforms. Normalization was performed against β actin: For: 5′ GACGACATGGAGAAGATCTG 3′ and Rev: 5′ TGAAGCTGTAGCCACGCTC 3′ which generated a 150 bp amplicon. Incorporation of SYBR Green dye into PCR products was monitored in real-time with a BIORAD iCycler detection system, allowing determination of the threshold cycle (C_T_) at which exponential amplification of PCR products begins. Each reaction was performed in triplicate. The ΔC_T_ values for PCR products were used to calculate the amount of mRNA amplified in each reaction.

### Atomic Iron Levels

Total extracts from neuronally differentiated SK-N-SH cells were analysed for Total Reflection X-ray Fluorescence, using Gallium as a reference standard at the concentration of 1 mg/ml. TXRF measurement was performed using a Bruker S2 total-reflection X-ray fluorescence spectrometer (Bruker, Germany). Data from three independent experiments were analyzed for total iron content.

### Transfection with small interfering RNAs

Double-stranded small interfering RNAs (siRNA) corresponding to homologous sequences of human (−)IRE DMT1 gene were designed with 3′-hydroxyl and two base overhangs on each strand (Qiagen). The two following gene-specific sequences were successfully used: (−)IRE DMT1 siRNA1: 5′- UCCGUGCUGCAUUGUAACUCA-3′, and (−)IRE DMT1 siRNA2: 5′-AGGCATTGCCAAAGAGCTTTA-3′. As a negative control (non-siRNA) was used the validated ALLstar Negative control (Qiagen). The buffered siRNAs were dissolved in RNAse free water to a final concentration of 20 µM. Cell transfection was carried out using HiPerfect Transfection Reagent (Qiagen) as follows: for 10 cm^2^ dishes, 4 µg of (−)IRE DMT1 siRNA or non-siRNA were incubated with 12 µl HiPerfect Transfection Reagent for 15 min at room temperature, according to the manufacturer's instructions. The transfection complex was added to SK-N-SH cells in 1 ml DMEM without serum and antibiotics. It was replaced with complete DMEM 24 hours later. Time-course studies were performed to assess the selective silencing of (−)IRE DMT1 protein in nuclear extracts. The OGD experiments were performed 40 and 30 hours after transfection for DMT1 siRNA1 and siRNA2, respectively.

### Chromatin Immunoprecipitation assay

Chromatin immunoprecipitation (ChIP) assays were performed using a ChIP assay kit (#9003S, Cell Signaling Technology, Massachusetts, USA). Briefly, primary coltures of mouse cortical neurons, exposed to 3 hours OGD followed by 2 hours reoxygenation, were cross-linked with 1% formaldehyde for 10 minutes at 37°C, the reaction was then stopped with a glycine solution was added for 5 minutes at room temperature. The cells were washed with ice-cold PBS, pooled, pelleted, and incubated on ice for 10 minutes in lysis buffer supplemented with 100 mM phenylmethylsulfonyl fluoride (PMSF), dithiothreitol (DTT), and a protease cocktail inhibitor mix. Nuclei were pelleted and resuspended in buffer supplemented with DTT, digested by micrococcal nuclease, and homogenized on ice. After centrifugation, sheared chromatin was incubated with anti-acetyl Histone H3 (Lys9/18) (#07-593, Upstate-Millipore, Massachusetts, USA), anti-RelA (#sc-372, Santa Cruz Biotechnology, Santa Cruz, CA, USA), or anti-IgG, as negative control, overnight at 4°C. Then, magnetic-coupled protein G beads were added and the chromatin was incubated for 2 hours in rotation. An aliquot of chromatin not incubated with antibodies was used as the input control sample. Antibody-bound protein/DNA complexes were washed, eluted, treated with proteinase and subjected to Real-time polymerase chain reaction (qRT-PCR) analyses. Immunoprecipitated DNA (4 µl) was amplified in 25-µl reaction mixture containing SYBR Green master mix (12,5 µl BIORAD). The primer specific for the mouse 1B/DMT1 promoter were as follows: For: 5′ ATGGGCGGAGCCTCCGTTCC 3′, and Rev: 5′ TCCATATCCCAGGAGCCAGC 3′, which generated a 130 bp amplicon. Incorporation of SYBR Green dye into PCR products was monitored in real-time with a BIORAD iCycler detection system, allowing determination of the threshold cycle (C_T_) at which exponential amplification of PCR products begins. Each reaction was performed in triplicate. The C_T_ values for PCR products were used to calculate the amount of chromatin immunoprecipitated in each reaction. Relative enrichment over the no-antibody INPUT and IgG negative control was first determined for each sample. Relative factor recruitment in OGD was then normalized to the control for each antibody.

### Reporter gene assay

In order to evaluate mouse 1B/DMT1 promoter activity during OGD, cortical neurons were transfected at 10 DIV using LF 2000 Reagent with 0.2 mg/well of the DMT1-pGL3 plasmid, kindly provided by J.A.Roth [38], and 0.8 mg/well of RelA, RelA-K310R or RelA-K310Q mutant constructs or empty expression vector pSG5 as negative control, as previously described [46]. To normalize the transfection efficiency, 0.02 µg/well *Renilla* luciferase control plasmid (Promega) was used. After 24 hours, neurons were exposed to 3 hour OGD as described above. Cells were then harvested and firefly and *Renilla* luciferase were measured by using Dual Luciferase Reporter Assay (Promega).

### Expression plasmids, transfection and treatments

The wild-type RelA plasmid, kindly provided by P.Jalinot [48], was used as template for the substitution of Lys 310 to Arginine or Glutamine in the mutant constructs RelA-K310R, as already described [42], and RelA-K310Q, respectively. The divalent metal transporter(−)IRE cDNA was obtained by PCR amplification from the (−)IRE DMT1 pDEST30 construct, kindly provided by M.Garrick [49] (FOR: 5′-CACTATAGATCTATGGTGTTGGATCCTAAAGAAAAGATGCCA-3′; REV: 5′-CGAATTAGATCTTCATCTGGACACCACTGAGTCAGCATCC-3′) and ligated into the BglII cloning site of pSG5 expression vector (Agilent Technologies, Stratagene products Division, La Jolla, CA, USA). After isolation of the sense clone by restriction analysis, the open reading frame was completely sequenced and the derived plasmid named 1B/(−)IRE DMT1 -pSG5. Transfection of differentiated SK-N-SH cells was carried out according to the manufacturer's instructions with Lipofectamine 2000 Reagent (LF2000, Invitrogen Corporation, Carlsbad, CA, USA). The day before transfection cultures were changed to normal growth medium containing serum and without antibiotics. Cells were transfected with expression plasmids encoding wild-type RelA, RelA-K310R or RelA-K310Q for 24 hours, before undergoing the OGD experiments, as previously described [42]. Treatment with Deferoxamine (DFO), dissolved in PBS, was performed at the concentration of 100 µM during OGD and overnight recovery. Cells transfected with pSG5 or 1B/(−)IRE DMT1-pSG5 were then incubated for 1 hour in Hepes buffer at pH 6.0, after washes at pH 7,4, with or without 100 µM ferrous iron, freshly prepared in water and kept on ice [49]. LDH release was determined as neurotoxicity index.

### Statistics

All results were calculated as the means ± se. The unpaired student's *t* test was applied to analyze differences between the groups. All differences were considered statistically significant at the *P* value<0,05. Statistical analyses were performed using Graph Pad Prism software (version 4.0).

## Results

### 1B/(−)IRE DMT1 is up-regulated during OGD exposure in neuronally differentiated SK-N-SH cells and in the cortex of a mouse model of tMCAO

We examined DMT1 expression in neuronally differentiated human neuroblastoma SK-N-SH cells exposed to OGD, already characterised as a cell-based model of brain ischemia [42], to establish the OGD-dependent modulation of DMT1.

**Figure 1:**
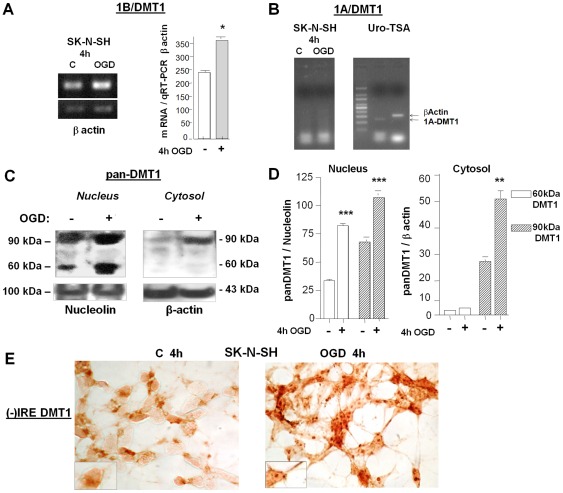
1B/(−)IRE DMT1 isoform is up-regulated in neuronal ischemia. (**A**) Semi-quantitative RT-PCR of the expression of 1B/DMT1 (222 bp) or β actin (312 bp) in neuronal SK-N-SH cells exposed to 4 hours of OGD. Data from densitometric analyses are expressed after normalisation of the cDNA in qRT-PCR for β actin. The 1B/DMT1 alternative transcript was significantly induced after 4 hours of OGD, with respect to controls. Bars are means ± s.e.m. of three separate experiments. *p<0.05 vs. relative control value. (**B**) Semi-quantitative RT-PCR of 1A/DMT1 (291 bp) in neuronal SK-N-SH exposed to 4 hours of OGD. The 1A/DMT1 transcript was undetectable in neuronal SK-N-SH both in control or OGD-treated cells. As a positive control, 1A/DMT1 transcript was amplified by semi-quantitative RT-PCR from the immortalised human urothelial cell line, URO-TSA. The results were analysed against β-actin (312 bp) as an internal control. These data were evaluated in at least three separate experiments. (**C**) Representative immunoblot of pan-DMT1 reactivity of nuclear and cytosolic extracts of differentiated human SK-N-SH cells exposed to 4 hours of OGD. The reactivity of pan-DMT1 significantly increased after OGD in the nuclear fractions, where only the 1B/(−)IRE DMT1 isoform is localised, and in the cytosolic extracts. (**D**) Densitometric analysis of pan-DMT1 reactivity was expressed as a ratio to relative nucleolin and β actin levels. Data are means ± s.e.m. of three separate experiments run in duplicate, ***p<0,001 and **p<0,01 vs. relative control values. (**E**) Neuronal SK-N-SH cells were exposed to 4 hours of OGD, fixed and immunostained for (−)IRE DMT1. The OGD-induced (−)IRE DMT1 reactivity was significantly enhanced after 4 hours of OGD, both in the nucleus and in the cell body.

We first investigated whether DMT1 alternative transcripts were up-regulated at the early phase of ischemic damage in neuronal cells. We performed semi-quantitative RT-PCR of the 5′ alternative 1A and 1B/DMT1 mRNAs. We found a significant increase of 1B/DMT1 mRNA at 4 h of OGD with respect to control cells (Fig. 1A) (*, p<0,05). However, 1A/DMT1 mRNA was not detectably expressed in the same samples ( Fig. 1B, left panel), in agreement with previous evidence of a highly restricted expression pattern for 1A/DMT1 in the kidney and duodenum [25]. 1A/DMT1 mRNA was amplified in the immortalised human urothelial cell line Uro-TSA, which was used as a control template (Fig. 1B, right panel).

We then investigated DMT1 protein levels during the early OGD phase in neuronal cells. The analysis was performed by western blots of cytoplasmic and nuclear extracts of SK-N-SH cells with a DMT1 antibody directed against epitopes common to the 4 isoforms (pan-DMT1). DMT1 protein was significantly up-regulated in both the nuclear compartment (***p<0,001) and in the cytosolic fraction (**p<0,01) by almost 50% in neurons exposed to 4 h of OGD, compared to control neurons. The pan-DMT1 antibody reactivity (Fig. 1C and D) revealed that both DMT1 components, with molecular masses of 60 and 90 kDa, were up-regulated in the nuclear extracts. These components are known to correspond to 1B/(−)IRE DMT1 [50], [51]. Only the predominant 90 kDa component was up-regulated in the cytosolic fractions, where 1B/(+/−)IRE DMT1 could co-localise [50], [51]. The increase of the (−)IRE DMT1 isoform, which is unresponsive to post-transcriptional regulation by intracellular iron levels through the IRE-IRP system, was confirmed by immunocytochemistry in SK-N-SH cells after 4 h of OGD. Fig. 1E clearly showed up-regulation of (−)IRE DMT1 in both nuclear and cytosolic cellular compartments after 4 hours of OGD. When staining was performed using the pan-DMT1 antibody, which did not discriminate between (+)IRE and (−)IRE isoforms, only a minor reactivity was detected (not shown), supporting the predominant up-regulation of (−)IRE isoforms during the early OGD phase.

**Figure 2:**
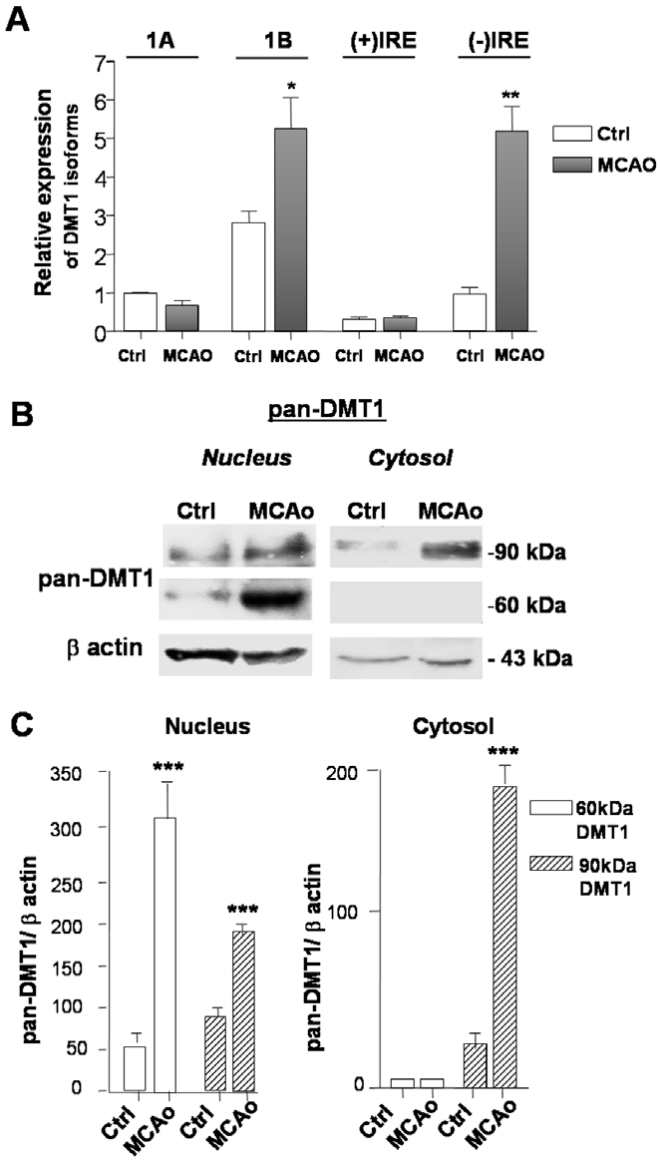
1B/(−)IRE DMT1 is up-regulated in ischemic cortices of mice exposed to tMCAO. (**A**) Gene expression analysis of the differentially spliced DMT1 isoforms by qRT-PCR in ischemic brain cortices of mice exposed to 1 hour transient MCAO followed by 1 hour reoxygenation compared with contralateral hemispheres (n = 3 per group). 1B/DMT1 and (−)IRE isoforms were significantly induced after MCAO. Values are expressed as fold change relative to 1A/DMT1 amplified in control (contralateral) hemisphere after normalization against β-actin cycles. Error bars represent ΔC_T_ means±s.e.m of two independent experiments performed in triplicate, *p<0,05, **p<0,01. (**B**) Representative image of pan-DMT1 immunoreactivity in nuclear and cytosolic extracts from brain cortices of mice exposed to 20 min of transient MCAO or from contralateral hemisphere (n = 3 per group). Nuclear extracts were prepared 4 h after the end of the experimental condition. The pan-DMT1 reactivity increased in brain extracts of mice exposed to MCAO, both in the nuclear and in the cytosolic fractions. (**C**) Densitometric analysis of pan-DMT1 reactivity. Values are expressed as ratios relative to β-actin levels. Error bars represent means ± s.e.m. of three separate experiments, ***p<0.007 vs. corresponding control values.

Data indicated that 1B/(−)IRE DMT1 expression increased at both the mRNA and the protein levels during the early post-ischemic phase. These results were supported by experiments in brains of mice subjected to 20 min MCAO, a condition causing a cortical infarct of 30±28 mm^3^ in mice evaluated three days after ischemic injury [43]. Examination of mRNA and protein in the ischemic cortical tissue confirmed a relevant DMT1 induction. Real time PCR of cortices exposed to MCAO and 1 hour reperfusion, showed increased expression of the DMT1isoforms 1B- and (−)IRE, when compared to relative contralateral cortices (Fig. 2A). Expression of 1A/DMT1 as well as (+)IRE DMT1 were much lower and unresponsive to the ischemic insult. Western blot analysis of brain extracts with the pan-DMT1 antibody confirmed a significant up-regulation of (−)IRE DMT1 in both nuclear and cytosolic compartments of ischemic cortices, respect to the relative contralateral hemispheres (Fig. 2B and C).

### 1B/(−)IRE DMT1 over-expression induces intracellular iron uptake and promotes iron-dependent cell death

**Figure 3:**
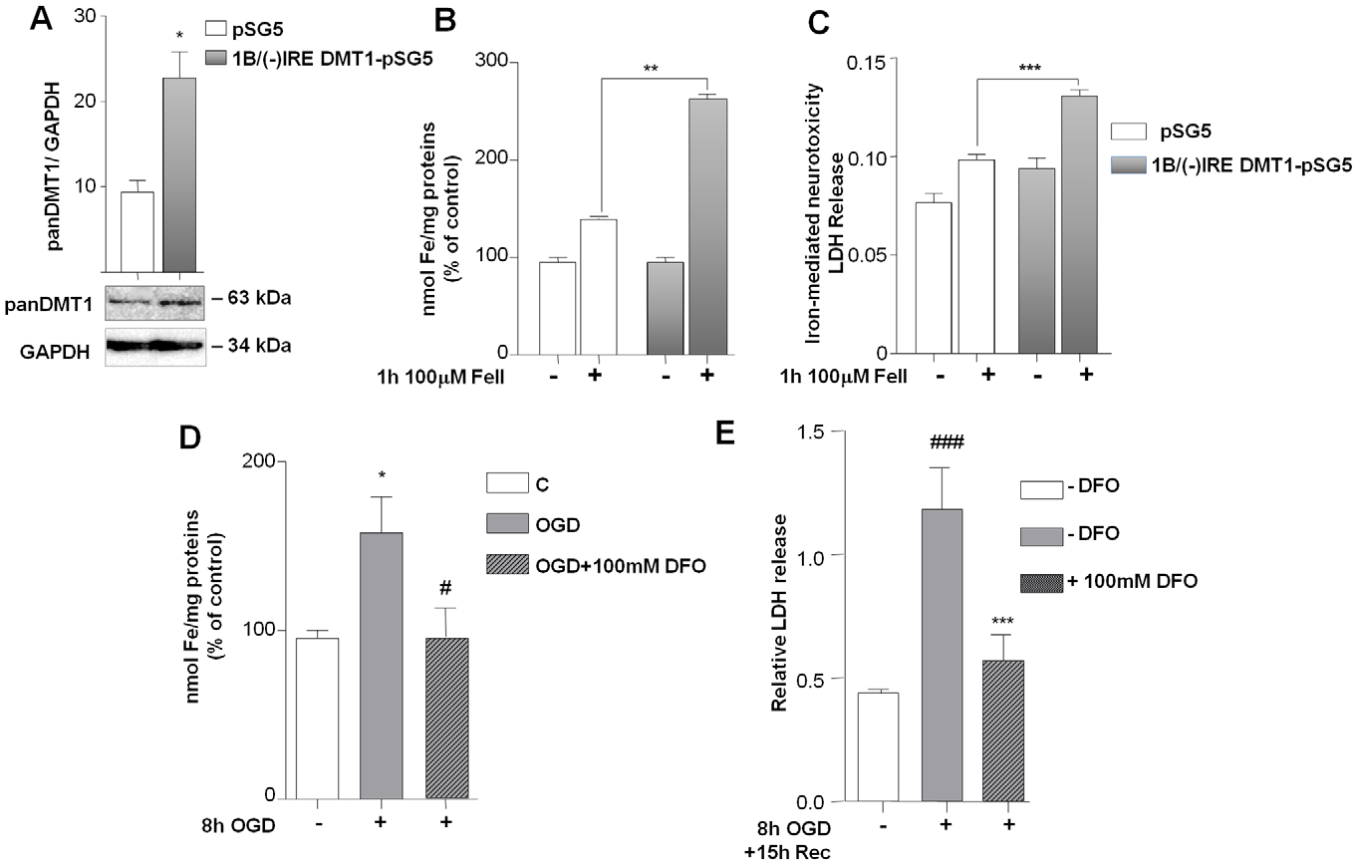
1B/(−)IRE DMT1 up-regulation enhances iron uptake and iron-dependent cell death. (**A**) Over-expression experiments were performed in differentiated SK-N-SH cells transfected for 24 hours with 1B-DMT1(−)IRE-pSG5 construct or pSG5 empty vector. Total cellular extracts were then analyzed for panDMT1 immunoreactivity after transfection and showed significant up-regulation of protein content. Densitometric analysis of pan-DMT1 immunoblots is expressed as ratio relative to GAPDH levels. Bars are the mean of three separate experiments. *p<0,01 vs pSG5 transfected cells. (**B**) The iron uptake induced by 1 hour exposure to 100 µM ferrous iron was higher in 1B/(−)IRE DMT1-pSG5 expressing cells than in pSG5 transfected cells. Total cell extracts were analyzed for atomic iron absorption (t-XRF). Atomic iron levels were expressed against gallium values, as an internal standard, and normalized to total protein concentration. Data, representing the mean±s.e.m. (percentage of control) of three independent experiments performed in triplicate.**p<0.001 vs. FeII treated pSG5. (**C**) The neurotoxicity elicited by 100 µM ferrous iron was higher in SK-N-SH cells over-expressing 1B/(−)IRE DMT1-pSG5 than in pSG5 expressing cells. Evaluation of neuronal injury was determined by LDH release assay. Data represent the mean±s.e.m. of three independent experiments performed in triplicate. ***p<0,0005 vs. FeII pSG5. (**D**)The total iron level significantly increased in neuronal SK-N-SH cells after 8 hours exposure to OGD. Data are means ± s.e.m. (percentage of control) of three independent experiments performed in triplicate. *p<0.05 vs. Control, *^#^*p<0,05 vs control OGD. (**E**) Cell survival was measured in neuronal SK-N-SH cells exposed to 8 hours of OGD followed by 15 hours of reoxygenation. Treatment with 100 µM DFO was performed during OGD and reoxygenation phase. Iron chelation with DFO significantly prevented OGD and reoxygenation-dependent cell death. Bars are mean±s.e.m. of three separate experiments, performed in triplicate. ^###^p<0.0003 vs. control; ***p<0.0001 vs. OGD in untreated cells.

To investigate the capability of 1B/(−)IRE DMT1 to uptake ferrous iron in our cell model, we ectopically expressed this isoform in differentiated human neuroblastoma cells. Cells transfected with the 1B/(−)IRE DMT1-pSG5 expression plasmid had increased levels of the DMT1 protein (Fig. 3A) (*p<0,01 vs pSG5 control). When compared to control cells, the 1B/(−)IRE DMT1 over-expressing cells exposed for 1 h to 100 µM ferrous iron elicited a higher iron uptake. This was shown by t-XRF analysis of the cellular iron content (Fig. 3B) (**p<0,005 vs pSG5 ferrous iron treatment). Moreover, the increase of iron uptake was associated with a significant increase of cell death (Fig. 3C) (***p<0,0005 vs pSG5 ferrous iron treatment).

### Endogenous expression of 1B/(−)IRE DMT1 during OGD promotes iron accumulation and cell death

To investigate the relevance of iron uptake in OGD-mediated cell death, we measured iron levels by t-XRF in cells exposed to 8 hours OGD. The intracellular iron level appeared to be increased in cellular extracts of differentiated human neuroblastoma cells exposed to 4 hours (data not shown) or 8 hours OGD (Fig. 3D). The co-exposure to 100 µM DFO totally abolished the iron uptake (Fig. 3E) (*p<0,05 vs.control; ^#^p<0,05 vs. control OGD). Moreover, cell death occurring in cultures exposed to 8 hours OGD and 15 hours of re-oxygenation, also appeared to be dependent on iron uptake as it was prevented by contextual application of 100 µM DFO (Fig. 3E) (^###^p<0.0001 vs. control; ***p<0.0001 vs. control OGD ).

**Figure 4:**
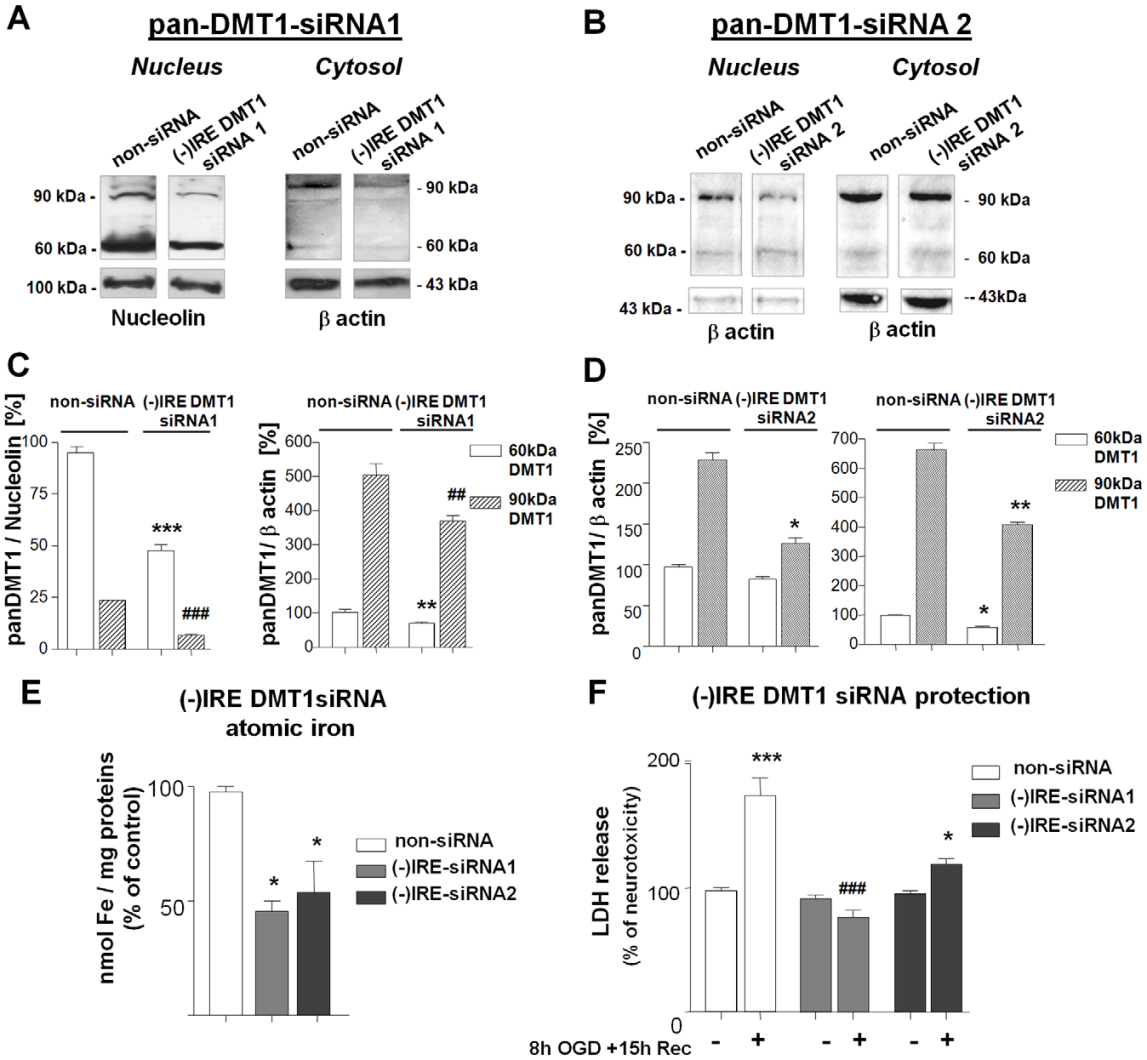
(−)IRE-DMT1 silencing decreases intracellular iron levels and prevents OGD neurotoxicity. Silencing experiments were performed in neuronal SK-N-SH cells transfected for 24 hours with control non-siRNA and (−)IRE DMT1 siRNA1(**A**) or (−)IRE DMT1 siRNA2 (**B**). Pan-DMT1 immunoreactivity was analysed 48 or 30 hours later in nuclear and cytosolic extracts and showed significant fewer (−)IRE DMT1 in siRNA-treated cells. (**C**) Densitometric analysis of pan-DMT1 immunoblots relative to nucleolin levels is expressed as the percentage of 60 kDa DMT1 values in Control non-siRNA treated cells. Bars are the mean±s.e.m. of three separate experiments. ***p<0.0001 and ^###^p<0.0001 vs. relative bands in non-siRNA. Densitometric analysis relative to β actin levels is expressed as the percentage of 60 kDa DMT1 values in control non-siRNA treated cells. Bars are mean±s.e.m. of three separate experiments. **p<0.005 and ^###^p<0.005 vs. relative non-siRNA controls. (**D**) Densitometric analysis relative to β actin levels is expressed as the percentage of 60 kDa DMT1 values in both nuclear and cytosolic extracts of Control non-siRNA treated cells. Bars are mean±s.e.m. of three separate experiments. *p<0.01 and **p<0.005 vs. relative non-siRNA controls. (**E**) Iron levels in neuronal SK-N-SH cells transfected with non-siRNA and (−)IRE DMT1 siRNA1 or (−)IRE DMT1 siRNA2 at the same time of OGD exposure. Total extracts were analyzed for atomic iron absorption (t-XRF). Data, representing mean±s.e.m. of three independent experiments performed in triplicate, are expressed as the percentage of non-siRNA control values. *p<0.05 vs. non-siRNA control. (**F**) Neuroprotection in neuronal SK-N-SH cells transfected for 24 hours with non-siRNA, (−)IRE DMT1 siRNA1 and (−)IRE DMT1 siRNA2. Cells were exposed to 8 hours of OGD and an additional 15 hours of reoxygenation. Neuronal injury was found with an LDH release assay. Silencing of (−)IRE DMT1 with both siRNA1 and siRNA2 specific oligonucleotides significantly prevented OGD-induced cell death. Data, representing mean±s.e.m. of three independent experiments performed in triplicate, are expressed as the percent of relative control values; ***p<0.001 vs. non-siRNA control; ^###^p<0,005 DMT1-siRNA1 OGD vs. non-siRNA OGD; ^*^p<0,05 DMT1-siRNA2 OGD vs. non-siRNA OGD .

We then investigated the effect of (−)IRE DMT1 silencing in OGD-treated neuronal cells. DMT1 protein expression was strongly reduced both in the nuclear fraction, where only the 1B/(−)IRE isoform is present, and in the cytosolic compartment of cells treated with either the (−)IRE DMT1 siRNA1 or siRNA2 (Fig. 4A–D). (−)IRE DMT1 knockdown significantly decreased intracellular iron levels in cultures treated with both siRNAs compared to non-siRNA treated cells (Fig. 4E) (*p<0,05 vs non-siRNA control). Furthermore, in the siRNA-treated cultures cell death induced by 8 hours of OGD and 15 hours of reoxygenation dramatically decreased (^###^ p<0,005 siRNA1 vs. non-siRNA OGD; *p<0,05 siRNA2 vs. non-siRNA OGD ) (Fig. 4F). These findings demonstrate that both reduced intracellular iron accumulation and upstream down-regulation of (−)IRE DMT1 expression can reduce OGD-mediated neuronal cell loss.

### OGD induced RelA binding and H3 histone acetylation on 1B/DMT1 promoter: effect of acetylated RelA at Lys310 on 1B/DMT1 transactivation

**Figure 5:**
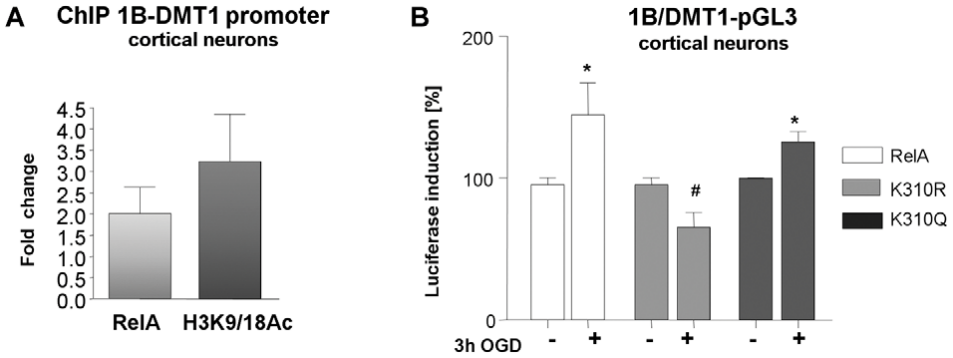
RelA activation of 1B/DMT1 promoter. (**A**) Chromatin Immunoprecipitation assay (ChIP) for mouse1B/DMT1 proximal promoter region showed increased binding of RelA subunit and acetylation of promoter-associated histone H3(K9/18) in cortical neurons exposed to 3 hours OGD, followed by 2 hours reoxygenation. Data are normalized against total chromatin DNA used for real time PCR reaction (Input) and IgG negative control determined for each reaction, respect to control samples. (**B**) Primary cortical neurons at 10 DIV were co-transfected with a mouse DMT1 luciferase reporter plasmid (m1B/DMT1-pGL3) and wild-type RelA or RelA-K310R or RelA-K310Q plasmids for 24 hours. The relative luciferase activity, normalized by Renilla luciferase, was measured after 3 hours of OGD. Wild-type RelA over-expression enhanced the OGD-induced DMT1 promoter activity, as well as the acetyl-mimic RelA-K310Q construct, while RelA-K310R over-expression significantly inhibited it. Bars are means ± s.e.m of three experiments run in triplicate. *p<0.05, vs. relative Control; ^#^p<0.05 vs. RelA OGD.

Since DMT1 has been proposed to be a NF-κB target gene [37], [38], we investigated the relationship between its expression and NF-κB activation during OGD. To establish the effect of OGD on endogenous NF-κB/RelA interaction with 1B-DMT1 promoter and the associated histone acetylation, chromatin immunoprecipitation analysis was performed using anti-RelA and anti-acetylated histone H3 (H3K9/18ac) antibodies. Results illustrated in Fig. 5A show that RelA binding to the NF-κB *cis*-acting element on 1B-DMT1 promoter increased in cortical neurons exposed to 3 hours OGD and 2 hours reoxygenation. Concomitantly, the acetylation of H3 histone associated to 1B/DMT1 promoter increased, in line with the increased expression of 1B/DMT1 during OGD.

Similar experimental conditions were used to analyse the NF-κB-dependent activation of a mouse 1B/DMT1 promoter-luciferase construct (m1B/DMT1-pGL3) [38]. Experiments were performed in mouse cortical neurons co-expressing plasmids coding for wild-type RelA or RelA carrying a mutation from lysine 310 to arginine (RelA-K310R) or the acetyl-mimic mutant with glutamine substitution of Lysine310 (RelA-K310Q). Since arginine has the same polar side chain and charge as lysine but cannot be acetylated, RelA-K310R can be used as a molecular determinant of hypo-acetylated RelA, while the glutamine residue, with uncharged, polar side chain can reproduce lysine 310 acetylation [52]. We found that OGD significantly induced 1B/DMT1 promoter activity in cells over-expressing the wild-type RelA and the RelA-K310Q, but not in cells expressing the RelA-K310R mutant plasmid (*, p<0,05 vs. relative control; #,p<0,01vs. RelA OGD) (Fig. 5B). These results highlight that NF-κB-mediated 1B/DMT1 transcription during OGD is dependent on Lys310 acetylation of RelA.

### Acetylation of RelA on Lys310 regulated DMT1 in neuroblastoma cells exposed to OGD

**Figure 6:**
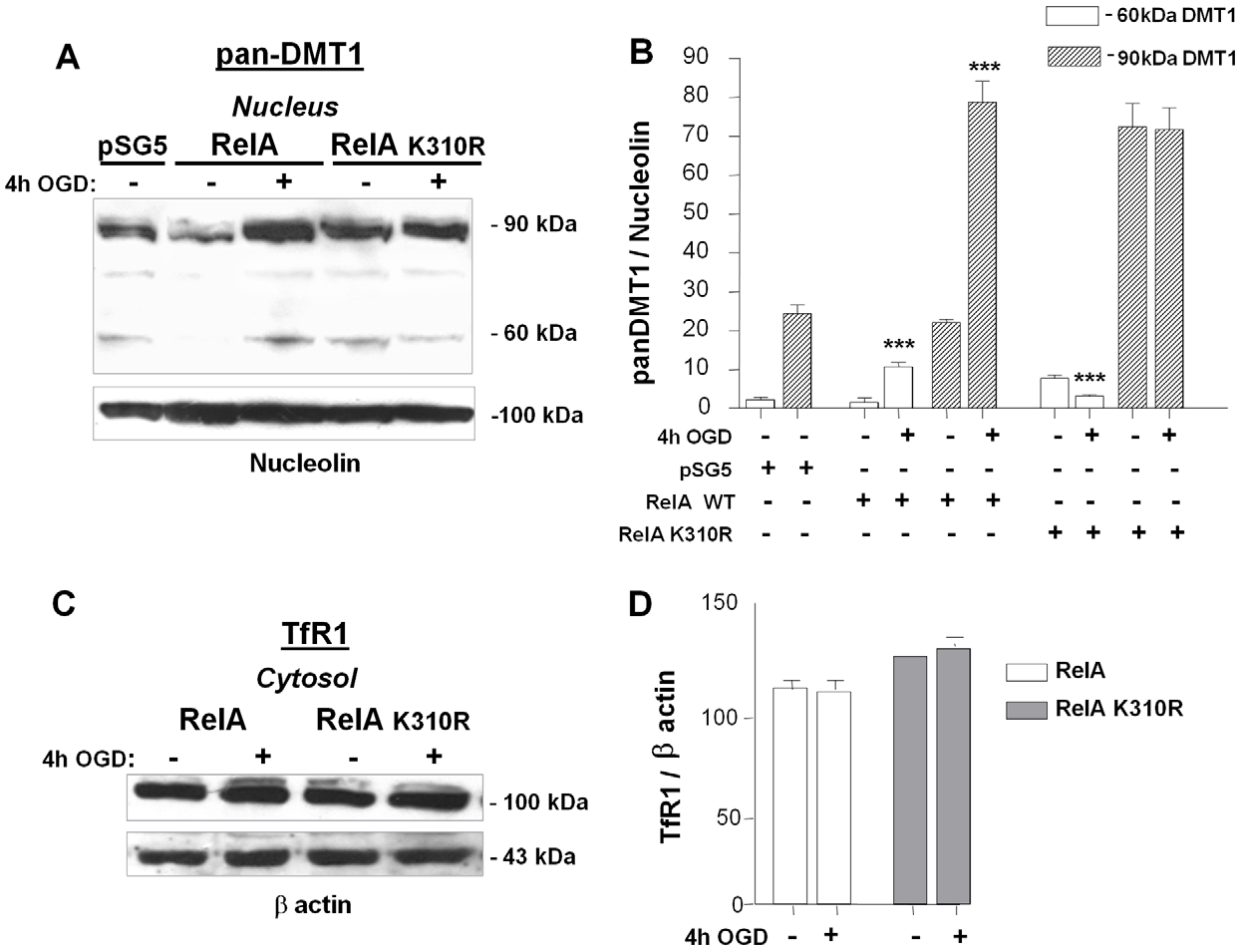
1B/(−)IRE DMT1 mediates the NTBI iron transport during OGD through Lys310-acetylated RelA. (**A**) Pan-DMT1 immunoreactivity in nuclear extracts from neuronal SK-N-SH cells transfected with wild-type RelA or RelA-K310R plasmids for 24 hours and further exposed to 4 hours of OGD. OGD produced a significant up-regulation of (−)IRE DMT1 protein in wild-type RelA overexpressing cells, with basal levels of DMT1 analogous to the empty vector (pSG5) transfected cells. Expression of RelA-K310R down-regulated the immature, partially glycosylated 60 kDa DMT1 isoform and failed to up-regulate the fully glycosylated 90 kDa DMT1 component. (**B**) Densitometric analysis of a representative pan-DMT1 immunoblots relative to nucleolin levels. Data from densitometric analysis of anti-panDMT1 antibody are expressed as as ratio relative to Nucleolin levels . Bars are the mean ± s.e.m. of three separate experiments (***p<0,0001 vs corresponding control value). (**C**) Immunoblot with anti-TfR antibody of cytosolic extracts from neuronal SK-N-SH cells transfected with wild-type RelA and RelA-K310R plasmids for 24 hours and exposed to 4 hours of OGD. The TfR protein level was not significantly modified in the early OGD phase in either RelA or RelA-K310R overexpressing cells. (**D**) Data from densitometric analysis of anti-TfR antibody immunoblots are expressed as a ratio relative to β-actin levels. Bars are means ± s.e.m. of three separate experiments.

We already showed that the vulnerability of neuronally differentiated SK-N-SH cells to OGD depends on NF-κB/RelA activation and acetylation at Lys310 [42], [43]. The cultures were transiently transfected with expression plasmids coding for wild-type RelA, the hypo-acetylated mutant RelA-K310R or the hyper-acetylated RelA-K310Q mutant. As previously reported [43], expression of RelA did not modify *per se* the cell survival, but greatly enhanced the cell vulnerability to OGD when compared to cells expressing the pSG5 empty vector. A similar cell death enhancement was produced by the hyper-acetylated RelA-K310Q mutant (Table 1). Conversely, the susceptibility of cultured cells to OGD was abolished by the expression of the RelA-K310R, as summarised in Table 1. Twenty-four hours after transfection, 1B/(−)IRE DMT1 up-regulation was examined by immunoblot analysis of nuclear extracts in cells exposed to OGD for 4 hours. The nuclear translocation of RelA and its acetylation on Lys310 were also verified by immunoblot analysis for all of the constructs tested (data not shown). In the cells over-expressing wild-type RelA, OGD promoted a significant up-regulation of both the 60 and 90 kDa components of nuclear DMT1, known to correspond to the 1B/(−)IRE isoform (Fig. 6A and B), as well as in cells over-expressing RelA-K310Q mutant (data not shown) (***p<0,0001 vs relative control). On the contrary, expression of RelA-K310R down-regulated the immature, partially glycosylated, 60 kDa DMT1 isoform to a level lower than basal value and failed to up-regulate the fully glycosylated 90 kDa DMT1 component (Fig. 6A and B). This presumably is a result of the rapid metabolic response of the endo H sensible-component [28], here associated with the regulated expression.

**Table 1:** SK-N-SH cell death after OGD depends on RelA-Lys310 acetylation.

	Reative LDH release	
	C	OGD15h
**pSG5**	0,05±0,005	0,27±0,02
**RelA**	0,04±0,003	0,45±0,04*
**RelA-K310R**	0,06±0,002	0,13±0,01#
**RelA-K310Q**	0,04±0,003	0,4 0±0,04*

Data show the cell survival of SK-N-SH cells over-expressing a control vector, pSG5, or wild-type RelA or RelA-K310R or RelA-K310Q constructs and exposed to 15 hours of OGD, compared to controls. Wild-type RelA and RelA-K310Q over-expression significantly enhanced toxicity due to OGD, while RelA-K310R over-expression prevented cell loss. Data are mean±s.e.m of three separate experiments run in triplicate and represent the amount of LDH released by each well, relative to total releasable LDH.

* p<0,0005 RelA or RelA-K310Q OGD vs relative pSG5;

# p<0,0005 RelA-K310R OGD vs relative RelA.

In the same experimental conditions, Transferrin Receptor, the other major receptor responsible for intracellular iron transport known to be induced during the late phase of post-ischemic reperfusion in the liver [53], was not involved in the early phase of OGD (data not shown) and RelA activation. Immunoblot analysis of the cytosolic extracts of neuronal cells transfected with wild-type RelA or RelA-K310R showed no change in the relative TfR component (Fig. 6C and D). Thus, the NTBI pathway through DMT1 seems to play a major role in the model of ischemia-associated neurodegeneration.

## Discussion

Iron has a conserved role in evolution as a finely coordinated iron and oxygen equilibrium is essential for aerobic cellular life. Iron was selected in molecular evolution because it is critical for oxygen transport, electron transfer and, in the central nervous system, neurotransmitter biosynthesis [1]. A finely regulated iron homeostasis is crucial for most brain activity and conditions affecting this equilibrium may cause neurological and cognitive dysfunction [54], [2]. However, despite the recognised importance of iron delivery to the brain, the mechanisms responsible for cerebral iron uptake and their regulation in the pathogenesis of ischemia remains to be established.

This study analyses the role of DMT1, responsible for non transferrin bound iron transport (NTBI), in cell-base and animal models of post-ischemic neurodegeneration. Our main finding was that 1B/(−)IRE DMT1 is a target gene for the Lys310-acetylated form of RelA during brain ischemia. The neuronal cell death induced by acetylation of RelA in brain ischemia was prevented by DMT1 silencing and iron chelation. The involvement of NTBI iron transport via DMT1 in neurodegenerative diseases has already been highlighted by studies that showed increased levels of iron and the DMT1 protein in SN dopaminergic neurons of both PD patients and PD animal models [29]. These studies showed that microcytic mice (mk/mk) and a Belgrade rat model carrying a G185R mutation of DMT1, which impairs iron transport, were more resistant to MPTP- and 6-hydroxydopamine-induced neurotoxicity, respectively. This evidence shows that neurotoxin-induced cell death is influenced by DMT1 iron transport and for this reason modulation of iron overload through DMT1 could be a new therapeutic strategy for PD treatment.

More recently, increased DMT1 expression, with consequent increased iron content in the brain, was shown in a cerebral ischemia rat model [55]. Pharmacological neuroprotection by tanshinone IIA, previously reported to be a natural NF-κB inhibitor [40], was associated with DMT1 down-regulation [13]. A pivotal role of DMT1 in brain ischemia is evident, as DMT1 is a proton co-transporter with an optimum ferrous iron uptake at pH 5.5 [49], and the ischemia-associated extracellular acidosis may very well contribute to exacerbated iron uptake via DMT1. Analysis of 1B/ and 1A/DMT1 transcripts in SK-N-SH cells revealed a selective expression of the 1B/DMT1 isoform that significantly was up-regulated during OGD exposure. This agrees with evidence that there is little 1A-DMT1 expression in the brain and when there is, it is restricted to the kidney and duodenum [25]. The up-regulation of 1B/(−)IRE DMT1 was evident even at the protein level during OGD.

Using immunoblot analysis with the pan-DMT1 antibody, we recognised two components with molecular masses of 60 and 90 kDa corresponding to the partially and fully glycosylated mature forms of DMT1, respectively [28]. After OGD, we found a specific increase of the protein in the nuclear compartment, where only the 1B/(−)IRE DMT1 isoform has been reported to localise [49], [50], [51]. A different reactivity was present in the cytosolic fraction, where both (−)IRE and (+)IRE isoforms could contribute to the single 90 kDa band detected here. The predominant contribution of (−)IRE DMT1 in the early phase of OGD was confirmed by immunocytochemical staining, using an antibody selective for (−)IRE DMT1 isoform. After 4 hours of OGD, the up-regulated (−)IRE protein displayed a nuclear and cytosolic distribution. This does not exclude the involvement of the (+)IRE isoform in the later phase of OGD. Indeed, immunocytochemistry with the pan-DMT1 antibody at 4 hours of OGD did not show the same relevant staining as the (−)IRE antibody, but displayed a stronger reactivity in the whole cell after 10 hours of OGD, when (−)IRE reactivity decreased (data not shown).

We then evaluated the changes of DMT1 expression in ischemic cortices of mice subjected to transient MCAO compared to the contralateral hemispheres. We found significant up-regulation of the 1B/(−)IRE DMT1 mRNA, but not of 1A/DMT1 isoform. Also, (+)IRE DMT1 expression appeared unchanged, possibly as a consequence of its negative post-transcriptional regulation by iron increase [25]–[27]. Along with 1B/(−)IRE DMT1 mRNA expression, 1B/(−)IRE DMT1 protein level raised in ischemic cortices. The DMT1 nuclear and cytosolic reactivity seemed to be able to be superimposed to that observed in the cellular model. This would suggest that 1B/(−)IRE DMT1 can contribute to the increase of cerebral iron content observed in mice models of brain ischemia [13]. Indeed, increases in intracellular iron content and cell death where detected along with the up-regulation of 1B/(−)IRE DMT1 in cultures exposed to OGD as well as in cells over-expressing 1B/(−)IRE DMT1 and exposed to ferrous iron. The application of iron chelator DFO during the OGD and reoxygenation phases, reduced both iron uptake and cell death, in accordance with previous reports showing the ability of DFO to suppress neuronal injury in experimental models of brain ischemia and subarachnoid hemorrhage [14], [56]–[60]. The DFO-induced reduction of intracellular iron uptake during OGD confirms the relevant role of iron chelation in the neuroprotection elicited by DFO. A possible stabilization of HIF-1α protein, regulated via the Fe^2+^- and O_2_-dependent enzyme prolyl hydroxylase (PDH), may contribute to DFO neuroprotection [61].

To interfere with the upstream pathway of intracellular iron transport, we performed (−)IRE DMT1 silencing. The increase of intracellular iron levels and cell death after OGD were reduced in siRNA treated cells, compared to non-siRNA treated controls. These results further confirm that (−)IRE DMT1 up-regulation and the subsequent increase of iron influx, greatly contributing to the development of acute ischemic neurotoxicity. Consistent with our results, previous findings showed that (−)IRE DMT1 silencing protected cortical neurons from L-DOPA-mediated cell death [31]. DMT1-dependent iron uptake and toxicity was specifically addressed in a recent study of DMT1 over-expression in the human neuroblastoma SH-SY-5Y stable cell lines, which demonstrated significant enhancement of iron uptake and associated cell death [62]. Furthermore, the up-regulation of (−)IRE DMT1, but not (+)IRE, was shown to induce iron accumulation in MPP^+^-treated MES23.5 dopaminergic cells [10], [39], [63] as a mechanism downstream to NF-κB activation.

A recent study has demonstrated that during brain ischemia, the aberrant activation of NF-κB p50/RelA driving the pro-apoptotic transcription of the Bim gene, relies on both p50/RelA nuclear translocation and RelA site-specific acetylation on Lys310 residue. Molecular and pharmacological deacetylation of RelA-Lys310, the latter mediated by the sirtuin-1 activator resveratrol, represses NF-κB-dependent activation of the Bim promoter as well as neuronal cell loss [43]. We therefore hypothesise that 1B/(−)IRE DMT1 up-regulation during OGD may depend on transactivation mediated by acetyl-RelA Lys310. The over-expression of wild-type RelA, as well as the acetyl-mimic construct RelA-K310Q, increased the 1B/DMT1 promoter activity, while expression of the acetylation-resistant RelA-K310R downregulated both. The peculiar role of endogenous NF-κB/RelA in the activation of 1B/DMT1 promoter through its proximal *cis*-acting element was established by chromatin immunoprecipitation analysis in cortical neurons exposed to OGD. The ischemic injury significantly increased the endogenous NF-κB/RelA binding to the promoter and the acetylation of the promoter-associated histone H3, supporting for a mechanism of chromatin regulation responsible for the RelA-dependent transactivation of 1B/DMT1. The same mechanisms, however, did not affect the expression of the transferrin receptor. The TfR was not responsive in early OGD phases, even after RelA over-expression, agreeing with previous reports linking TfR activation to the late phase of ischemia and reperfusion [53].

In conclusion, this study shows that site-specific acetylation of RelA at Lys310 switches on the NF-κB-mediated transcription of 1B/(−)IRE DMT1 during the early phase of brain ischemia. Further studies will be addressed to establish whether 1B/(−)IRE DMT1 may become a druggable target for treatment of post-ischemic brain injury, likewise the Lys310-acetylated RelA [43]. Interfering with 1B/(−)IRE DMT1 function may be the strategy of choice to selectively block the iron-dependent onset of neurodegeneration without perturbing iron uptake mediated by other mechanisms at the basis of the physiological aerobic homeostasis.
